# Acute Administration of Natural Honey Protects Isolated Heart in Normothermic Ischemia 

**Published:** 2012

**Authors:** Afshin Gharekhani, Moslem Najafi, Hamed Ghavimi

**Affiliations:** a *Department of Clinical Pharmacy, Faculty of Pharmacy, Tabriz University of Medical Sciences, Tabriz, Iran.*; b *Biotechnology Research Center, Tabriz University of Medical Sciences, Tabriz, Iran.*; c *School of Pharmacy, Tabriz University of Medical Sciences, Tabriz, Iran.*; d *Student Research Committee, Faculty of Pharmacy, Tabriz University of Medical Sciences, Tabriz, Iran. *

**Keywords:** Natural honey, Acute administration, Normothermic ischemia, Arrhythmia, Infarct size

## Abstract

This study intended to assess the efficacy of acute administration of natural honey on cardiac arrhythmias and infarct size when it is used during the normothermic ischemia in isolated rat heart.

During 30 min of regional normothermic ischemia followed by 120 min of reperfusion, the isolated hearts were perfused by a modified drug free Krebs-Henseleit solution (control) or the solution containing 0.125, 0.25, 0.5 and 1% of freshly prepared natural honey (test groups), respectively. Cardiac arrhythmias were analyzed and determined through the recorded ECGs. The infarct size was measured using computerized planimetry package.

At the ischemic phase, honey (0.25 and 0.5%) decreased the number and duration of ventricular tachycardia (VT), total number of ventricular ectopic beats (VEBs), duration and incidence of reversible ventricular fibrillation (VF) and total VF (p < 0.05 for all). During the reperfusion, concentrations of 0.125, 0.25 and 0.5% lowered the number of VT (p < 0.05), duration of reversible VF (p < 0.01) and total number of VEBs (p < 0.05). In addition, VT duration was reduced significantly with honey 0.125 and 0.25%. Moreover, the infarct size was 45.6 ± 3.4% in the control group, while the perfusion of honey (0.125, 0.25 and 0.5%) reduced it to 14.8 ± 5.1 (p < 0.001), 24.6 ± 7.3 (p < 0.01) and 31.4 ± 7.3% (p < 0.05), respectively.

Regarding the results, it is concluded that the acute administration of natural honey in normothermic ischemia conditions can protect the rat heart as the reduction of infarct size and arrhythmias. Conceivably, the antioxidant and free radical scavenging activity, the reduction of necrotized tissue and the providence of rich energy source are more important mechanisms in cardioprotective effects of natural honey.

## Introduction

Natural honey has been used for different medicinal purposes since ancient times. Human use of honey is traced to some 8000 years ago as shown by Stone Age paintings (1). Ancient Egyptians, Assyrians, Chinese, Greeks and Romans employed honey for wounds and diseases of the intestine (2). In addition to the important role of natural honey in the traditional medicine, during the past few decades, it was subjected to the laboratory and clinical investigations by several research groups. The most remarkable discovery was the antibacterial activity of honey that has been mentioned in numerous studies (3, 4). Natural honey exhibits bactericidal activity against many enteropathogenic organisms, including those of the *Salmonella *and *Shigella *species, and enteropathogenic *E. coli *(5, 6). Besides, it has been reported that honey has a restraining influence on the growth of some methicillin-resistant *Staphylococcus aureus *strains (4). The precise composition of honey varies according to the plant origin on which the bee feeds. However, all honeys almost contain flavonoides (such as apigenin, pinocembrin, kaempferol, quercetin, galangin, chrysin and hesperetin), phenolic acids (such as ellagic, caffeic, p-coumaric and ferulic acids), ascorbic acid, tocopherols, catalase (CAT), superoxide dismutase (SOD), reduced glutathione (GSH), Maillard reaction products and peptides, most of which work together to provide a synergistic antioxidant effect (6-10). On the other hand, honey is well known for its advantages within the wound environment. It maintains a moist wound environment that promotes healing and its high viscosity helps to provide a protective barrier to prevent the infection. In addition, the mild acidity and low-level hydrogen peroxide release both aid tissue repair and contribute to the antibacterial activity of honey (11). The effectiveness of honey to treat the severely infected cutaneous wounds was also confirmed in recent clinical case studies (12). Honey has been reported to have immunomodulatory activities (13). In one study, it was demonstrated that honey modulates the activity of monocytic cells to repair the wounded tissue by releasing anti-inflammatory cytokines and growth factors (14). Additionally, in an experimental model of inflammatory bowel disease, the oral intake of honey had anti-inflammatory effects in rats (15). In another study, Al-Waili and Boni demonstrated anti-inflammatory effects of honey in humans after ingesting 70 g of it. They showed that the consumption of honey lowered the mean plasma concentrations of thromboxane B2, PGE2 and PGF2α by 48%, 63% and 50%, respectively (16). In addition to the mentioned anti-inflammatory mechanisms, honey has also been reported to inhibit the activities of cyclooxygenase-1 and cyclooxygenase-2 that shows the supplementary anti-inflammatory effects (17). Honey, interestingly, has shown to prevent the reactive oxygen species (ROS)-induced low density lipoprotein (LDL) oxidation *in-vitro *studies and therefore exhibits the beneficial cardiovascular protection (18, 19). To date, the majority of studies have focused on the potential health benefits of honey for human, but its efficacy on cardiovascular diseases such as arrhythmia and myocardial infarction is not completely understood. In 2008, for the first time we showed cardioprotective effects of ischemic preconditioning with honey in the setting of regional ischemia when it was used 10 min before the ischemia initiation to 10 min after it (20). Recently, we also reported the protective effects of chronic oral administration of natural honey (45 days) against the ischemia/reperfusion (I/R) injuries (21). However, in the present study, the role of acute administration of natural honey on cardiac arrhythmias and infarct size, when used for the whole period of 30 min normothermic ischemia followed by 120 min reperfusion, was investigated in an isolated rat heart.

## Experimental

*Chemicals*

The following chemicals were purchased: Honey (as wax free and freshly prepared 2 months before the beginning of the study, from Oskou, East Azerbaijan, Iran), Triphenyltetrazolium chloride (TTC) (Sigma), Evans blue dye, Formalin, NaCl, NaHCO3, KCl, KH2PO4, MgSO4, CaCl2, D-glucose, D-fructose (Merck Company), Sodium pentobarbital (Kela Company, Belgium) and Heparin (Darupakhsh Company, Iran).

*Animals and surgical procedure*

Male Sprague-Dawley rats (weighing 270-320 g) were used in this study. Subjects were pretreated with intraperitoneal (IP) injection of 300 IU heparin and then, anaesthetized with sodium pentobarbital (50-60 mg/Kg, IP) (22). The hearts were excised rapidly and mounted on a non-recirculating langendorff apparatus under the constant pressure of 100 mmHg at 37°C and perfused throughout the experiments with modified Krebs-Henseleit (K/H) solution which was freshly prepared and equilibrated with 95% O2-5% CO2. A latex fluid filled balloon was inserted into the left ventricle and inflated to give a preload of 8-10 mmHg (22-24). After 20 min of stabilization, the hearts were subjected to 30 min regional normothermic ischemia (by temporary occlusion of left anterior descending coronary artery) followed by 120 min of reperfusion. In the control group (n = 8), the hearts were perfused only by normal K/H solution throughout the experiment, while in the test groups (4 groups, n = 8-10 in each group), they were perfused with K/H solution containing 0.125, 0.25, 0.5 and 1% of honey during I/R. Regarding the existence of high amounts of energy sources in the composition of honey (fructose and glucose) and in order to investigating the probable roles of those sugars, the effects of equivalent value of fructose and glucose (~38 and 30 g per 100 g of honey, respectively) was added to the K/H solution then studied in separate groups at the same conditions. An epicardial ECG was recorded continuously by a physiograph during the experiment. Based on the Lambeth conventions, the ECGs were analyzed to determine the total number of ventricular ectopic beats (VEBs), the number of beats occurring as ventricular tachycardia (VT), the incidence and duration of VT and ventricular fibrillation (VF) during the ischemia and the first 30 min of reperfusion time (23, 25, 26).

*Measurement of myocardial infarction size*

To determine the infarct size, at the end of 120 min reperfusion, the ligature around the left anterior descending coronary artery was retied and the hearts were slowly perfused with 2-3 mL of saline solution containing 0.25% of Evans blue dye (w/v) via the side arm of aortic cannula (22, 24, 25). The hearts were frozen and then, the ventricles of the frozen hearts were sliced transversely in a plane perpendicular to the apico-basal axis into 2 mm thick sections. The slices were then incubated with 1% (w/v) TTC solution in phosphate buffer for 15 min at 37°C to dye the non-infarcted region (24, 27). This procedure resulted in the normally perfused tissue being stained blue, non-infarcted, non-perfused tissue stained brick red and infarcted tissue remaining unstained and appeared pale (24, 28, 29). The reported experiments were carried out in accordance with the Guide for the Care and Use of Laboratory Animals (National Institutes of Health Publication No. 85-23, revised 1985).

*Statistical analysis*

Except for the incidence of VT and VF which are expressed as percentage, all the other results are expressed as mean ± SEM. To compare the number of VEBs, VT and duration of VT and VF between the groups, the Mann-Whitney non-parametric U-test was employed. For analyzing the incidence of VT and VF, the Fisher exact test (Chi-square with Yates correction) was used. The mean percentage of infarct size was analyzed using one-way ANOVA and then significant differences were examined through LSD post-hoc range test (21, 24, 29). Differences between groups were considered significant at a level of p < 0.05.

## Results and Discussion

The effects of acute administration of natural honey, glucose and fructose on normothermic ischemia and reperfusion arrhythmias are summarized in Tables 1 and 2. During the ischemia, honey (0.25 and 0.5%) significantly lowered the number, the duration and the incidence of recorded arrhythmias compared to the control group (p < 0.05). In addition, except for the number of VEBs and ischemic VT, honey 1% had significant reduction effect on duration and the incidence of VT and reversible VF (p < 0.05). Perfusion of glucose and fructose in the individual test groups produced marked reduction in number, duration and incidence of ischemic VT (p < 0.01 for all).

**Table 1:** Effects of natural honey (0.125-1%), glucose (0.3%) and fructose perfusion (0.38%) during 30 min regional ischemia on the ischemic arrhythmias

**Groups**	**Ischemic arrhythmias**							
	**VEBs number**	**VT number**	**VT duration (sec)**	**Rev VF duration (sec)**	**Rev VF incidence (%)**		**Total VF incidence (%)**	**VT incidence (%)**
**Control**	568 ± 84	111 ± 22	18 ± 4	44 ± 27	60	60		100
**Honey (0.125%)**	354 ± 131	145 ± 119	18 ± 15	14 ± 12	37.5	37.5		75
**Honey (0.25%)**	226 ± 94**	32 ± 16*	5 ± 2**	2 ± 2*	12.5*	12.5 *		50*
**Honey (0.5%)**	278 ± 47*	38 ± 20*	6 ± 3*	0*	0*	0*		75
**Honey (1%)**	346 ± 160	104 ± 100	10 ± 10*	0*	0 *	0*		25 *
**Glucose**	361 ± 104	5 ± 4***	1 ± 1***	167 ± 110	25	25		25**
**Fructose**	512 ± 113	8 ± 6**	1 ± 1***	1 ± 1*	12.5	12.5		25**

*** p < 0.001, ** p < 0.01, * p < 0.05 *vs. *control. N = 8-10 in each group. VT: Ventricular Tachycardia, VEBs: Ventricular Ectopic Beats (Single + Salvos + VT), Rev VF: Reversible Ventricular Fibrillation, Total VF (Incidences of Rev VF + Irreversible VF).

**Figure 1:**
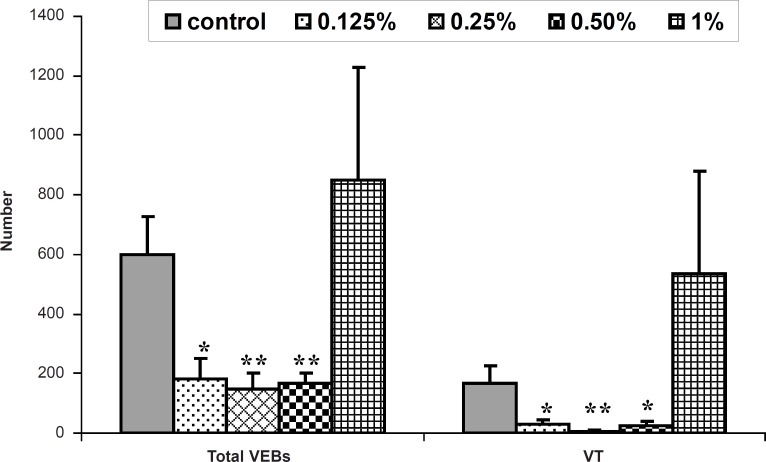
The total number of ventricular ectopic beats (VEBs) and ventricular tachycardia (VT) during 30 min reperfusion in the control and isolated rat hearts receiving 0.125-1% of honey-enriched K/H solution. ** p < 0.01, * p < 0.05 *vs. *control group (n = 8-10 in each group

During the reperfusion time, the perfusion of 0.125% of honey-enriched K/H solution significantly reduced the number of VEBs and VT and the time spent in reversible VF and VT (p < 0.05). Moreover, the duration and the incidence of reversible VF and total VF showed significant reduction by honey 0.25% (p < 0.01). This concentration lowered total number of VEBs, number and duration of VT markedly (p < 0.01). As shown in Table 2, duration and incidence of reversible VF and total VF were reduced significantly by honey 0.5 and 1% (p < 0.01 for duration and p < 0.05 for incidence, respectively). Furthermore, as illustrated in Figure 1, honey 0.5% significantly reduced number of VEBs and VT (p < 0.01 and p < 0.05, respectively). Administration of glucose and fructose reduced total number of VEBs, number and duration of VT (p < 0.05 for both).

**Table 2:** Effects of natural honey (0.1251%-), glucose (0.3%) and fructose perfusion of (0.38%) during ischemia and reperfusion on the reperfusion phase arrhythmias

**Groups**	**Reperfusion arrhythmias**							
	**VEBs number**	**VT number**		**VT duration (sec)**	**Rev VF duration (sec)**	**Rev VF incidence (%)**	**Total VF incidence (%)**	**VT incidence (%)**
**Control**	601 ± 127	167 ± 59	28 ± 10		173 ± 86	70	70	80
**Honey (0.125%)**	182 ± 67*	28 ± 15*	3 ± 1*		16 ± 10**	50	50	50
**Honey (0.25%)**	145 ± 54**	5 ± 5**	1 ± 1**		0***	0**	0**	12.5*
**Honey (0.5%)**	166 ± 35**	25 ± 13*	5 ± 3		19 ± 19**	12.5*	12.5*	37.5
**Honey (1%)**	849 ± 378	534 ± 347	69 ± 46		1 ± 1***	12.5*	12.5*	37.5
**Glucose**	234 ± 59*	15 ± 8*	2 ± 1*		157 ± 109	25	25	37.5
**Fructose**	241 ± 70*	11 ± 7*	1 ± 1**		15 ± 15*	12.5*	12.5*	25

*** p < 0.001, ** p < 0.01, * p < 0.05 vs. control. N = 810- in each group. VT: Ventricular Tachycardia, VEBs: Ventricular Ectopic Beats (Single + Salvos + VT), Rev VF: Reversible Ventricular Fibrillation, Total VF (Incidences of Rev VF + Irreversible VF).

**Figure 2:**
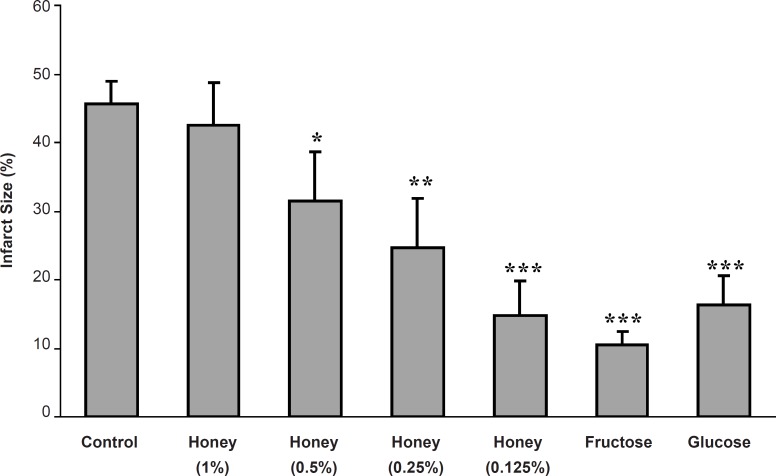
Myocardial infarct size as a percentage of risk zone volume in the control and isolated rat hearts receiving honey (0.125-1%), glucose (0.3%) and fructose (0.38%) during 30 min ischemia followed by 120 min reperfusion. *** p < 0.001, ** p < 0.01, * p < 0.05 *vs. *control group (n = 8-10 in each group).

In the control group, the infarct size was 45.6 ± 3.4% while the acute administration of 0.125, 0.25 and 0.5% of honey-enriched K/H solution significantly reduced the infarct size to 14.8 ± 5.1 (p < 0.001), 24.6 ± 7.3 (p < 0.01) and 31.4 ± 7.3% (p < 0.05), respectively. In addition, both fructose and glucose reduced the myocardial infarct size from the control value to 10.4 ± 2.1 and 16.3 ± 4.3%, respectively (p < 0.001 for both groups) (Figure 2).

Cardiac arrhythmias remain a major source of morbidity and mortality in developed countries (30). In cardiac surgery and myocardial infarction, ventricular arrhythmias such as VT and VF are the most important cause of mortality (31). Ischemic condition results in the inhibition of fatty acid metabolism then accumulation of their toxic metabolites in the heart. These molecules have been shown to be deleterious to the recovery of myocardial function of the reperfused heart (32, 33) and have been shown to be as a cause of ventricular arrhythmias (34). Myocardial I/R results in significant damage to the heart and this study was conducted to evaluate probable cardioprotective effects of acute administration of natural honey against I/R injuries in isolated rat heart. Our results demonstrated that honey can protect cardiac tissue against arrhythmias such as VEBs, VT and VF. During normothermic ischemia and reperfusion phases, perfusion of 0.25% of honey-enriched K/H solution significantly reduced the number, duration and incidence of VT. Additionally, the time spent in reversible VF and incidences of reversible VF and total VF was significantly reduced by 0.25, 0.5 and 1% of honey-enriched K/H solution**. **Although honey 0.125% showed significant anti-arrhythmic effects on the number and duration of reperfusion-induced arrhythmias, but this concentration had no significant effect on ischemia-induced arrhythmias. Regarding the results, it seems that concentrations of 0.25 and 0.5% were optimum to protect the ischemic-reperfused isolated rat hearts against arrhythmias in our model. However, honey (1%) had some non-significant proarrhythmic actions at the reperfusion phase. At the same time, the lowest concentration (0.125%) was not efficient to produce the significant anti-arrhythmic effects.

Although natural honey has been applied for medicinal purposes since ancient times (35); however, in the case of cardiovascular diseases, most of the previous studies were focused on honey›s effects against the cardiovascular risk factors such as hyperlipidemia and production of free radicals (36-40). The results of our previous work revealed that preischemic administration of natural honey (0.25, 0.5 and 1%) as a pharmacologic preconditioning agent had antiarrhythmic and cardioprotective activities in isolated rat heart (20). In addition, recently we showed that chronic oral administration of honey for 45 days had antiarrhythmic effect in rat (21). In spite of some methodological differences between the present and above studies (for example: administration period of honey), the results of current study are in consistent with the previous works. That is, acute, chronic and short time preischemic administration protocols of honey protect isolated rat heart against I/R-induced arrhythmias (20, 21). Moreover, similar to preischemic administration results, it seems that the low concentrations of honey are more effective than higher pro-arrhythmic actions used concentrations. Probably, the existence of high amount of glucose in higher concentrations of honey may change glucose to lactate in ischemic myocytes which in turn causes electrical and contractility disturbances in the heart (20).

As depicted in Tables 1 and 2, both fructose and glucose produced the marked anti-arrhythmic effects as a reduction of number and duration of VT. This finding may suggest that fructose and glucose, as the most constituents of natural honey, may play a pivotal role in the anti-arrhythmic effects of natural honey. When the anti-arrhythmic effects of fructose and glucose in ischemic time were compared to their effects at reperfusion phase, both agents showed more significant effects on ischemia-induced arrhythmias. It was proposed that during the ischemia, due to the lack of oxygen supply and restricted mitochondrial oxidation of fatty acids and carbohydrate, the exogenously administered fructose or glucose likely act to regenerate the depleted resources of ATP production and consequently improve cardiac hemodynamic functions. Indeed, during a mild to moderate ischemia, the rates of glucose uptake and glycolysis become accelerated which may provide an important source of ATP to maintain the optimal control of membrane ion flux. In addition, increasing the translocation of glucose transporters to the sarcolemmal membrane of myocytes may well increase the rate of glycolysis and provide some benefits to the ischemic heart. As a result, it appears that providing an adequate amount of glucose may be an effective approach to mitigate the myocardial ischemic injuries. However, it should be noted that during severe ischemia, high glycolytic rates may actually contribute to the ischemic injury, secondary to the production of glycolytic products such as lactate and H+ (41). Conversely, during the myocardial reperfusion, there is an overshoot in the rate of fatty acid oxidation and impaired pyruvate oxidation and accelerated nonoxidative glycolysis. High rates of fatty acid *ß*-oxidation considerably restrain the glucose oxidation which in turn leads to a marked imbalance between the glycolysis and glucose oxidation. This uncoupling is a major source of the net H+ production in the heart which can exacerbate the reperfusion injuries. If glycolysis is coupled to glucose oxidation, H+ production from glycolysis is zero (42). Additionally, it seems that fructose, like glucose, may contribute to myocardial energy production. Evidence from various investigations suggests that ROS contribute to the pathophysiology of myocardial I/R injury. ROS, which are formed within the ischemic myocardium and in the first moments of reperfusion, are known to be cytotoxic to surrounding cells and cause lethal arrhythmias. In addition to the ROS, ionic imbalance (such as calcium overload) and impaired electrical activity are believed to trigger severe ventricular arrhythmias. Therefore, the inhibition of the ROS production can be an important strategy for the treatment of ventricular arrhythmias (43, 44). There are both enzymatic and nonenzymatic defense mechanisms in the heart that protect the myocardial tissue against the harmful effects of ROS. The former includes GSH, ascorbic acid, *α*-tocopherol and *β*-carotene, while the latter consists of SOD, CAT, glutathione peroxidase (GPx) and glutathione reductase (GR) (9). The heart normally possesses sufficient activities of these antioxidant components. However, under the conditions of I/R that weaken the cardiac antioxidant system and make the tissue overexposed to ROS, the myocardium may be subjected to the oxidative damage (45). Therefore, the antioxidant activity of honey and the scavenging of free radicals demonstrated in some previous studies may play an important role in the above protective effects of honey as well (15, 19, 46-49). In general, the antioxidant capacity of honey appeared to be a result of the combined activity of a wide range of compounds including phenolics, peptides, organic acids, enzymes, Maillard reaction products, and possibly other minor components like antioxidant vitamins and flavonoides (10, 49). As a result, it may be suggested that protective effects of natural honey against the I/R-induced arrhythmias may also be related to its both enzymatic and nonenzymatic antioxidants (7-9). Moreover, honey is extraordinarily rich in minerals, mainly calcium, potassium, chlorine, sodium, iron, magnesium and zinc (6, 9, 50). It seems that some of these minerals may be partially responsible for the anti-arrhythmic effects of honey. It has been reported that zinc has an inhibitory action on the free radical formation in the heart, since it interferes with the processes that initiate arrhythmias. Magnesium may prevent cardiac arrhythmias through inhibiting the voltage-dependent calcium channels. Moreover, sodium, through the process of Na+/ Ca2+ exchange, can prevent the increase of Ca2+ ions within the cardiac cells and reduce the incidence of ventricular arrhythmias (9).

**Figure 3:**
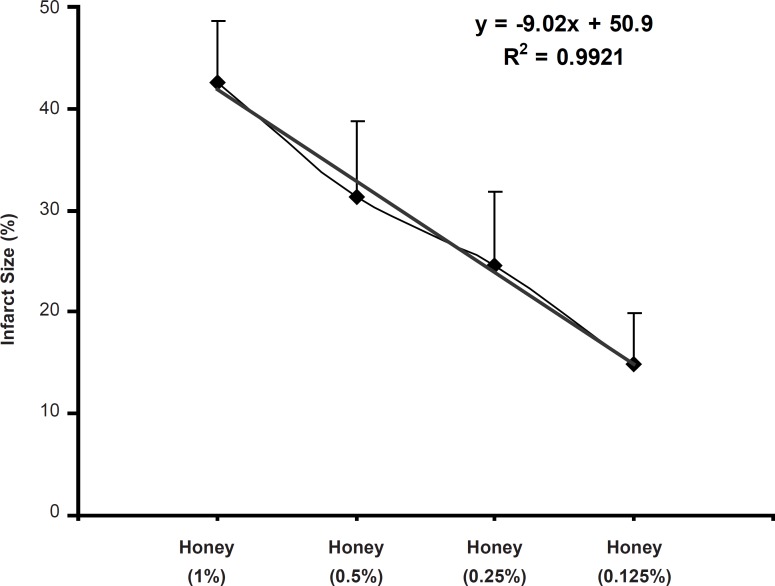
Relationship between the myocardial infarct size (%) and honey concentrations (0.125-1%) applied during I/R in the isolated rat hearts (n = 8-10 in each group)

In the present study, the acute administration of natural honey also caused significant cardioprotection against the myocardial infarction (Figure 2). Similar to the anti-arrhythmic effects, the reduction of infarct size in this work is in consistence with the findings of previous studies. In 2008, it was showed that the perfusion of isolated rat heart with 0.25, 0.5 and 1% of honey, 10 min before the ischemia to 10 min after it, produced clear and marked reduction in infarct size in rat hearts (20). Regarding the used concentration range of honey in our model, it seems that the reduction of infarct size is concentration-dependent and there is a reverse linear relationship (with an equation of y = - 9.02 x + 50.9 and r2 = 0.992) between the honey concentration and its cardioprotective effect (Figure 3). 

Therefore, the lower concentrations of honey (in particular 0.125 and 0.25%) are more effective than higher concentrations for decreasing myocardial infarction. Additionally, as a probable mechanism, maybe natural honey in higher concentrations, like some anti-arrhythmic drugs, not only cannot produce cardioprotective actions but also it may lead to deleterious effects via inadequate metabolism of its carbohydrate content and consequently a rise in lactic acid level and then acidosis in myocytes. Similar to the anti-arrhythmic effects, fructose and glucose may probably have important role in the efficacy of natural honey on myocardial infarction due to providing rich energy source. As discussed previously, since there are limited evidences about effectiveness of honey against the I/R-induced injuries, the exact cardioprotective mechanisms of natural honey are not clear. However, the antioxidant and free radical scavenging activity of natural honey have been shown in some previous *in-vitro *and *in-vivo *studies (10, 15, 49). Hence, we proposed that the antioxidant properties of honey may partially reduce the infarct size in ischemic reperfused rat heart. Other potential mechanisms to decrease the myocardial infarct size through honey may include anti-inflammatory effects (2, 16, 51), anti-arrhythmic effects (20) and decrease in the area of necrotized tissue (14, 16).

By considering the data, it may be concluded that the acute administration of honey at normothermic ischemia conditions can protect the isolated rat hearts and consequently has antiarrhythmic activity and reducing infarction size. The existence of rich energy sources, many vitamins, minerals, enzymes and antioxidant and radical scavenging activity, may probably involve in the cardioprotective effects of natural honey in such conditions. Future studies are required to determine other pharmacological benefits and the exact protective mechanism (s) of honey in ischemic-reperfused condition.
